# Framing Public Opinion on Physician-Patient Conflicts on Microblog: A Comparative Content Analysis

**DOI:** 10.3389/fpubh.2022.831638

**Published:** 2022-02-04

**Authors:** Wanqi Gong, Qin Guo

**Affiliations:** ^1^School of Journalism and Communication, Guangdong University of Foreign Studies, Guangzhou, China; ^2^School of Humanities, Zhejiang University of Technology, Hangzhou, China

**Keywords:** physician-patient relationship, opinion leader, framing, microblog, COVID-19

## Abstract

**Introduction:**

Physician–patient conflicts in China have increased more than ten times from the 2000s to the 2020 and arouse heated discussions on microblog. The outbreak of the COVID-19 pandemic is believed to have brought a turnaround in the physician–patient relationship. However, little is known about the similarities and differences among the views of opinion leaders from the general public, physicians, and media regarding physician–patient conflict incidents on microblog, and whether the outbreak had an impact on this.

**Objective:**

This study aims to explore how opinion leaders from the physicians, general public, and media framed posts on major physician–patient conflict incidents on microblog, and compare the microblog post frames before and after the COVID-19 pandemic. The findings will provide more objective evidence of the attitudes and perspectives of the health professionals, general public, and media on physician–patient conflicts, and the influence of pandemics on physician–patient relationship.

**Methods:**

A comparative content analysis was conducted to examine the posts (*n* = 941) of microblog opinion leaders regarding major physician–patient conflicts in China from 2012 to 2020.

**Results:**

Post-pandemic microblog posts used more cooperation, positive and negative frames, but mentioned less health-related knowledge; no difference was found in the use of conflict and attribution frames. Results on the use of frames by opinion leaders from different communities found that the media used more conflict, cooperation, attribution, and positive frames, but used fewer negative frames and mentioned less health-related knowledge than general public and physicians. Results on the use of frames for different incidents found that incidents of violence against physicians used more cooperation, positive and negative frames and mentioned less health-related knowledge; in the contract, incidents of patient death used more attribution frames and mentioned more health-related knowledge.

**Conclusion:**

The physician and general public opinion leaders share some similarities in their post frames, implying that no fundamental discrepancy between them regarding physician–patient conflict incidents. However, the imbalanced use of frames by media microblogger would cultivate and reinforce the public perception of physician–patient contradictions. After the COVID-19 pandemic, more cooperation and positive frames were used in the posts, indicating an improvement in the physician–patient relationship in China.

## Introduction

Physician–patient conflicts have increased more than ten times from the 2000's to the 2010's in China (1) and have a substantial effect on physician–patient mistrust and relationship (2). Nie et al. (2) found that intense physician–patient conflicts increased the physicians' defense, further exacerbated physician–patient communications, and produced poorer health outcomes and negative news reports, finally leading to more serious physician–patient mistrusts and conflicts. Many factors account for the poor patient–physician relationship in China, including the complicated medical system, limited medical resources compared to the large population, and high medical costs (3). These factors lead to limited patient encounter time and insufficient physician–patient communication, which further cause dissatisfaction and even serious conflicts between physicians and patients (3, 4).

Social media offers an optional channel for physician–patient communication. Compared to face-to-face and online e-health service communications, microblog provide a more open, comfortable, and relatively equal platform for physician–patient communication (5, 6). When communicating on a microblog, patients are usually not in an emergency situation, and physicians are less stressed as they are away from their workplace. It is more valuable in Asian contexts since online communication can reduce patient's inhibitions of expressing their concerns and emotions in face-to-face situations and could possibly strengthen physician–patient communication (7). Therefore, an increasing number of physicians and patients worldwide have turned to microblog to communicate, disseminate, and discuss health-related issues (8, 9).

Opinion leaders refer to people who influence other's opinions or attitudes on social issues (10), including health education and promotion (11). Microbloggers can be verified by microblog platforms as health professionals, media, or celebrities. Some of these verified microbloggers have attracted millions of followers on Sina Weibo. Recent studies have found that these verified microbloggers have the ability to disseminate information and share their views on social issues with their numerous followers; therefore, they act as opinion leaders on social media (12). Opinion leaders on microblog could affect public opinion regarding health topics and the adoption of healthy behaviors (13, 14), such as reinforcing the stereotypes of mental illness, tobacco use (15), and disease prevention (16). Han and Wang (13) found that verified microbloggers have higher connection scores (in-degree and out-degree) than non-verified microbloggers, and the top influential verified microbloggers hold central positions in the information flow process on health-related topics.

Physician–patient conflicts, especially violence incidents, have aroused heated discussion among microbloggers from various communities (physicians, the general public, and media) (17). It must be recognized that since the opinion leaders come from different backgrounds, they have distinct standpoints: physicians represent the health professional perspectives, the general public understands and perceives issues from a patient's perspective, and the media concentrates on physician–patient conflicts to promote audience interest and garner attention (18). The differentiated standpoints lead to different concerns and framing strategies. Lu et al. (7) found that different stakeholders have different concerns about the online health community: patients focused on topics related to lung cancer symptoms and diabetes drugs, caregivers were more concerned about topics related to lung cancer drugs, and patients expressed more emotions than caregivers and health professionals.

Framing theory points out that media reports shape public's understanding of news story through utilizing certain reporting frames (19). Within the social media context, message frames of opinion leaders also would define trending topic's emphasis and thereby influence public's interpretation and opinion of the topic (20, 21). Message frame refers to the speaker's structured reporting or presentation style, including viewpoints, words, and sentence patterns (19, 22). Nip and Fu (20) found that media microbloggers utilized more thematic frame on corruption issues than government, independent news sources, and other microbloggers; and have more emotional expressions than other types of microbloggers. It demonstrated that opinion leaders from different communities on social media utilize different message frames to discuss news and further influence their followers' interpretations and evaluations of a specific issue or incident (20). On health issue, different stakeholders also adopt different message frames to express their opinions on microblog, which contribute to public opinion regarding health topics, the patient's adherence to their physicians, and, ultimately, the effects of the prescribed treatment (17, 18, 22, 23).

Violence against physicians in China have aggravated since 2010. Lancet called for protecting Chinese doctors on January 2020 since “the attack scale, frequency and viciousness on Chinese doctors are particularly severe” (24). Chinese health workers behaved responsibly and even devotionally during the COVID-19 pandemic. Public also showed comprehension and appreciation for physicians according to the media reports (46, 48). It was hoped that the physician–patient relationship would improve since the COVID-19 pandemic (25); however, a few physician–patient conflicts still have been reported since 2020. Therefore, a comparison analysis of opinion leader's microblog posts on physician-patient conflicts before and after COVID-19 pandemic could provide empirical evidence for the change of public opinion and physician-patient relationship. Besides, previous studies have concentrated more on incidents of violence against physicians (26). However, patient death incidents and no death incidents causing physician–patient conflicts have also aroused public attention, such as the Yulin mother suicide incident (2017) and physician's selfies in the operating room (2014).

Previous studies on physician–patient communication and relationship mostly adopted survey or interview (27, 28), which might have self-report bias and cannot reflect the dynamic interaction process among different groups. Therefore, to systematically investigate different stakeholders' perspectives on physician–patient conflicts and the change before and after COVID-19 pandemic, this study tries to explore message frames of posts by opinion leaders from physicians, the general public, and media on major physician–patient conflicts on microblog from 2012 to 2020. Findings will extend our understanding of consensus and discrepancies between patients and physicians with respect to their cognitive roles, mutual expectations, and communication. This study will provide more objective evidence of the attitudes and perspectives of health professionals, the general public, and media on physician–patient conflicts through content analysis. Opinion leaders influence their followers' attitudes toward physician–patient topics, which may further affect physician–patient offline relationship and healthcare outcomes (2). Therefore, this study will also contribute to building a foundation for future studies on strengthening physician–patient communication, enhancing physician–patient relationship, and expanding health knowledge discussions on social media.

We focus on the frames that are applicable to physician–patient conflict incidents on microblog. Semetko and Valkenburg (31) defined the conflict, cooperation, responsibility, and valence frames (25), and these are still applicable in the social media context (17). The conflict frame captures audiences' attention by concentrating on conflicts among individuals and/or groups, whereas the cooperation frame focuses on cooperation among individuals and/or groups (29). Opinion leaders on microblog influence their followers' perceptions of reporting incidents as either cooperative or incompatible (i.e., in conflict) by utilizing the conflict or cooperation frame (30). The responsibility frame, which focuses on the responsibility attribution regarding an issue or incident (31), is used by opinion leaders or the media to promote the responsible aspects of a specific incidents (32, 33), such as physician–patient conflict incidents. The valence frame refers to the reporting of incidents in either positive or negative terms (34). Opinion leaders influence public judgment regarding an incidents or event as either good or bad using positive or negative frames, respectively (20, 35).

Besides message frames, this study also analyzes whether the message promoting health knowledge related to the incident opinion leaders on social media are found to be effective in promoting health knowledge and behavior (36). Physicians and media and opinion leaders may introduce health knowledge that is based on the discussed physician–patient incidents to promote medical knowledge among the public.

To investigate the differences in message frames used by opinion leaders from the health industry, general public, and media when expressing their opinions on physician–patient incidents, we framed the following research questions:

RQ1: How are (1) conflict, (2) cooperation, (3) responsibility, (4) positive frames, (5) negative frames, and (6) health promotion used in opinion leaders' posts regarding physician–patient conflicts on microblog?

RQ2: How do different opinion leaders' microblog posts about (1) conflict, (2) cooperation, (3) responsibility, (4) positive frames, (5) negative frames, and (6) health knowledge promotion differ in their use before and after the COVID-19 pandemic?

RQ3: What are the differences in the use of (1) conflict, (2) cooperation, (3) responsibility, (4) positive frames, (5) negative frames, and (6) health knowledge promotion among opinion leaders from the general public, physicians, and media?

RQ4: What are the differences in the use of (1) conflict, (2) cooperation, (3) responsibility, (4) positive frames, (5) negative frames, and (6) health knowledge promotion with respect to different physician–patient conflicts?

## Methods

A comparative content analysis was conducted to investigate the microblog posts of the opinion leaders from the general public, physicians, and media on physician–patient conflict incidents in China.

### Selection of Microblog Platform

The posts were collected from Weibo for several reasons. According to iResearch's report, Weibo is the largest Chinese microblog with 56.6% of the market share of active users and 86.6% of the market share with respect to browsing time based on data from China's 2010 microblog market (37). Since 2012, Weibo has required all users to register with real names to improve cyber security, and provided additional verified badges to users in public interest areas (e.g., health professions) to authenticate their practitioner status and enlarge their influence (e.g., more exposure and followers). Combining these features, this study focused on discussions regarding physician–patient incidents on Weibo.

### Selection of Physician–Patient Conflict Incidents

The study period ran from 2012 to 2020. The year 2012 was set as the starting time point because Weibo required all users to register with real names and provided additional verified badges, while 2020 was set as the end point so that the study could compare the differences of message frames regarding physician–patient conflict incidents before and after the pandemic. To make the results more convincing, two incidents were selected for the year 2020, the time point after the outbreak.

Physician–patient conflict incidents were selected through a survey pretest. First, the three most highly discussed physician–patient conflict incidents for each year were nominated based on media reports and online discussions (e.g., the database of Zhiweidata, Weibo trending). Subsequently, the participants in the pretest (*n* = 298) were asked to recall details of the incidents, and those incidents that were most clearly remembered were selected as the study cases for further analysis. This included (1) the fatal attack in Harbin Hospital (2012); (2) the fatal attack in Wenling Hospital (2013); (3) “selfies” taken by physicians in the operating room (2014); (4) physician fainting in the operating room (2015); (5) the Wei Zexi incident (2016); (6) the Yulin mother incident (2017); (7) the Peking University Hospital incident (2018); (8) the Civil Aviation General Hospital incident (2019); (9) the Beijing Chao-yang Hospital incident (2020–1); and (10) the Yanqing Hospital incident (2020–2) (Appendix 1). The incidents were further classified according to their consequences: (1) incidents of violence against physicians (2012, 2013, 2018, 2019, 2020–1, and 2020–2), (2) no death incidents (2014 and 2015), and (3) patient death incidents (2016 and 2017).

### Recruitment

The analysis unit was the Weibo posts that discussed the nominated physician–patient incident. Previous studies found that public discussions on Weibo have limited timeliness; public engagement reaches a peak within 5 days, then declines markedly, and almost stops within a week (22, 38). Therefore, we set the unit of time to seven days after the first exposure on Weibo. For non-criminal incidents, posts were collected for seven days after their first exposure on Weibo; for criminal incidents, the analysis time was extended by another seven days after the trial.

Eligible posts were obtained in three steps (see Appendix 2). First, preliminary collection. All Weibo posts that discussed the selected incidents were captured *via* (1) an existing database platform (Zhiweidata, one of the most complete and authoritative platforms for detecting, recording, and preserving the top-discussed incidents on multiple social media platforms in mainland China), or (2) crawler software (GooSeeker) using keyword searches (e.g., the names of the physician, patient, and hospital). Second, opinion leader selection. Information on the microbloggers who published these posts was collected. For each incident, the study selected the key opinion leaders based on the number of followers, incident-related posts, and retweeted posts and comments, then classifies them into three categories based on their practitioner status as verified by Weibo: media, physician, and general public, and finally selected the top three most influential Weibo users from these three categories as the opinion leaders for that incident. Since some microbloggers acted as opinion leaders in more than one incident, for instance, People's Daily was selected as the opinion leader in eight of the ten incidents. Therefore, a total of 55 opinion leaders were selected instead of 90 (3 most influential microbloggers × 3 account types × 10 incidents), including 12, 23, and 20 opinion leaders from the media, general public, and physicians, respectively. Third, final data collection. For each incident, we collected all posts that discussed the incident and were posted by the selected opinion leaders resulting in a total of 941 posts. By incident types: (1) incidents of violence against physicians, *n* = 661; (2) no death incidents, *n* = 67; and (3) patient death incidents, *n* = 213. By opinion leader types: (1) media opinion leader, *n* = 430; (2) general public opinion leader, *n* = 182; and (3) physician opinion leader, *n* = 329.

The large difference between the sub-sample sizes might be attributed to the incident's consequences and accompanying emotions. Weibo is a venue to not only browse information but also vent emotions (39). Death-related incidents are believed to result in a larger discussion on Weibo (22), because negative emotions arouse efficient information processing and subsequently enhance people's engagement (40).

### Coding

The coding scheme was developed based on previous studies (32) and was modified to match our specific research setting and research purposes. The initial coding scheme followed the generic framework in the literature, that is, conflict, responsibility, cooperation, and valence frames (positive and negative aspects of an event). Although these frames are well practiced in news reports, an increasing number of studies suggest that they can also be applied to social media (41). When discussing conflict incidents, Chinese netizen's concerns include not only the parties involved, but also the government and society, and tend to require official media and opinion leaders objectively inform and evaluate the incidents and guide the public to establish the right values. For physician–patient incident specifically, people incline to go beyond the incident and expand the discussion to the current situation of physician–patient relationship and how to improve it (42). Opinion leaders will increase the general public's understanding of physicians by promoting health literacy in hopes of improving the physician–patient relationship. Hence, promotion of health knowledge was also included in the coding scheme (43).

The conflict frame was constructed based on whether the microblog posts mentioned the disagreement between (1) patient or/and patient's family and physician; (2) patient and the public opinion; (3) physician and the public opinion; and (4) two or more sides in the patient/patient's families, physician, and public opinion involved in the incident.

The cooperation frame was operated based on whether the microblog posts mentioned the cooperation between patient/patient's family and physician in the specific incident, and whether it mentioned cooperation in the broader discussion of the physician–patient relationship. Four categories were built to reflect the different dimensions of physician–patient cooperation: (1) a good communication environment, (2) physician's efforts, that is, to better understand patient's concerns, (3) patient's efforts, that is, to well understand physician's suggestions, and (4) cooperation in other formats.

The attribution frame refers to the posts that are responsible for the specific incident, for instance, (1) society/government, (2) physician/hospital, and (3) patient/patient's family. The positive and negative frames judged whether the posts discussed the positive and negative sides, and each frame was adopted in all three categories. It should be noted that the positive and negative sides encompassed not only the evaluation of the nature of the incident, but also the outlook on the physician–patient relationship.

The promotion of health knowledge identified whether the posts mentioned medical knowledge relevant to the issue, and two categories were used to develop the frame: (1) scientific knowledge directly related to the incident (e.g., knowledge of the specific disease that causes the death of the patient), and (2) other scientific knowledge related to the incident (e.g., knowledge of painless labor in mother-related incidents) (see Tables 1a, b for details).

**Table 1a:** Overall landscape of the frames uses by opinion leader types (bootstrapping *n* = 2,000).

**Category**	**Coding description**	**Media**	**Public**	**Physician**	***F***	***p-*value**
		***n* (mean)**	***n* (mean)**	***n* (mean)**	***df* = 2**	
**Conflict**						
Physician-patient conflict	Disagreement between patient/patient's family and doctor	147 (0.34)^a^	17 (0.09)^b^	81 (0.25)^c^	21.648	<0.001
Patient-public disagreement	Disagreement between patient and the public opinion	11 (0.03)^a^	4 (0.02)^a^	9 (0.03)^a^	0.068	0.934
Physician-public disagreement	Disagreement between doctor and the public opinion	17 (0.04)^a^	18 (0.10)^b^	24 (0.07)^ab^	4.312	0.014
General conflict	Disagreement of two sides or to more than two sides of the problem or issue	77 (0.18)^a^	21 (0.12)^a^	45 (0.14)^a^	2.470	0.085
		(0.146)	(0.082)	(0.121)	Mean=0.125	
**Cooperation**						
Cooperation	Cooperation between patient and doctor	58 (0.14)^a^	5 (0.03)^b^	15 (0.05)^b^	14.735	<0.001
Communication	Good communication between patient/ patient's family and doctor	24 (0.06)^a^	3 (0.02)^b^	9 (0.03)^ab^	3.520	0.030
Physician's understanding	Patient's concerns are well understood by the doctor	17 (0.04)^a^	1 (0.00)^b^	7 (0.02)^ab^	3.150	0.043
Patient's understanding	Doctors' views are well understood by the patient	14 (0.03)^a^	4 (0.02)^a^	4 (0.01)^a^	1.790	0.182
		(0.066)	(0.018)	(0.027)	Mean = 0.043	
**Attribution**						
Government	Society/government has the responsibility to solve the problem	114 (0.27)^a^	40 (0.22)^ab^	55 (0.17)^b^	5.219	0.006
Physician	Doctors/hospital have the responsibility to solve the problem	47 (0.11)^a^	14 (0.08)^ab^	18 (0.06)^b^	3.701	0.025
Patient	Patient or family has the responsibility to solve the problem	48 (0.11)^a^	13 (0.07)^a^	28 (0.09)^a^	1.472	0.230
		(0.162)	(0.123)	(0.102)	Mean=0.134	
**Positive**						
Bright side	Emphasize the bright side of the case/issue	184 (0.43)^a^	25 (0.14)^b^	60 (0.18)^b^	42.023	<0.001
Advantage	General advantage or specific benefit of the case/issue for any side	134 (0.31)^a^	15 (0.08)^b^	34 (0.10)^b^	37.375	<0.001
Future benefit	Promising development or praise the current state of the parent-doctor relationship	156 (0.36)^a^	18 (0.10)^b^	25 (0.08)^b^	61.322	<0.001
		(0.367)	(0.107)	(0.255)	Mean=0.231	
**Negative**						
Dark side	Emphasize the dark side of the case/issue	75 (0.17)^a^	52 (0.29)^b^	119 (0.36)^b^	17.820	<0.001
Disadvantage	General disadvantage or specific cost of the case/issue for any side	95 (0.22)^ab^	30 (0.17)^a^	87 (0.26)^b^	3.316	0.037
Future cost	Problematic future development or criticize the current state of the parent-doctor relationship	74 (0.17)^a^	31 (0.17)^a^	87 (0.26)^b^	5.690	0.003
		(0.189)	(0.207)	(0.297)	Mean=0.230	
**Popular medical science**						
General knowledge	Scientific knowledge about the disease in the issue	4 (0.01)^a^	5 (0.03)^a^	9 (0.03)^a^	2.040	0.131
Specific knowledge	Issue-related knowledge	14 (0.03)^a^	31 (0.17)^b^	33 (0.10)^c^	17.552	<0.001
		(0.021)	(0.099)	(0.064)	Mean=0.051	

a, b, c, *different superscripts indicate the existence of a statistically significant difference*.

**Table 1b:** Overall landscape of the frames uses by opinion incident types (bootstrapping *n* = 2,000).

**Category**	**Coding description**	**Violence against physicians**	**No-death**	**Patient-death**	***F***	***p-*value**
		***n* (mean)**	***n* (mean)**	***n* (mean)**	***df* = 2**	
**Conflict**						
Physician-patient conflict	Disagreement between patient/patient's family and doctor	215 (0.33)^a^	0 (0.00)^b^	30 (0.14)^c^	28.464	<0.001
Patient-public disagreement	Disagreement between patient and the public opinion	8 (0.01)^a^	0 (0.00)^a^	16 (0.08)^b^	14.185	<0.001
Physician-public disagreement	Disagreement between doctor and the public opinion	23 (0.04)^a^	15 (0.22)^b^	21 (0.10)^c^	22.482	<0.001
General conflict	Disagreement of two sides or to more than two sides of the problem or issue	88 (0.13)^a^	9 (0.13)^ab^	46 (0.22)^b^	4.402	0.013
		(0.126)	(0.090)	(0.133)	Mean = 0.125	
**Cooperation**						
Cooperation	Cooperation between patient and doctor	71 (0.11)^a^	5 (0.08)^ab^	2 (0.01)^b^	10.406	<0.001
Communication	Good communication between patient/ patient's family and doctor	32 (0.05)^a^	2 (0.03)^ab^	2 (0.01)^b^	3.417	0.033
Physician's understanding	Patient's concerns are well understood by the doctor	25 (0.04)^a^	0 (0.00)^ab^	0 (0.00)^b^	5.486	0.004
Patient's understanding	Doctors' views are well understood by the patient	19 (0.03)^a^	3 (0.05)^ab^	0 (0.00)^b^	3.654	0.026
		(0.056)	(0.037)	(0.005)	Mean=0.043	
**Attribution**						
Government	Society/government has the responsibility to solve the problem	153 (0.23)^a^	4 (0.06)^b^	52 (0.24)^a^	5.630	0.004
Physician	Doctors/hospital have the responsibility to solve the problem	18 (0.03)^a^	10 (0.15)^b^	51 (0.24)^c^	54.724	<0.001
Patient	Patient or family has the responsibility to solve the problem	76 (0.12)^a^	0 (0.00)^b^	13 (0.06)^b^	6.575	0.001
		(0.125)	(0.070)	(0.182)	Mean=0.134	
**Positive**						
Bright side	Emphasize the bright side of the case/issue	226 (0.34)^a^	18 (0.27)^a^	25 (0.12)^b^	20.841	<0.001
Advantage	General advantage or specific benefit of the case/issue for any side	167 (0.25)^a^	3 (0.05)^b^	13 (0.06)^b^	25.323	<0.001
Future benefit	Promising development or praise the current state of the parent-doctor relationship	198 (0.30)^a^	1 (0.02)^b^	0 (0.00)^b^	58.019	<0.001
		(0.299)	(0.110)	(0.060)	Mean=0.231	
**Negative**						
Dark side	Emphasize the dark side of the case/issue	188 (0.28)^a^	3 (0.05)^b^	55 (0.26)^a^	9.131	<0.001
Disadvantage	General disadvantage or specific cost of the case/issue for any side	171 (0.26)^a^	5 (0.08)^b^	36 (0.17)^b^	8.570	<0.001
Future cost	Problematic future development or criticize the current state of the parent-doctor relationship	159 (0.24)^a^	1 (0.02)^b^	32 (0.15)^c^	12.305	<0.001
		(0.261)	(0.045)	(0.193)	Mean=0.230	
**Popular medical science**						
General knowledge	Scientific knowledge about the disease in the issue	11 (0.02)^a^	0 (0.00)^a^	7 (0.03)^a^	1.834	0.160
Specific knowledge	Issue-related knowledge	37 (0.06)^a^	13 (0.19)^b^	28 (0.13)^b^	12.167	<0.001
		(0.036)	(0.097)	(0.082)	Mean=0.051	

a, b, c, *different superscripts indicate the existence of a statistically significant difference*.

A yes–no binary coding strategy was used to indicate whether the posts included a particular framing item. The value of each frame was calculated by averaging the scores of the framing items. Two well-trained coders analyzed all the posts. When disagreements occurred, the authors and two coders collaboratively reviewed and discussed the posts to determine the content frames. The Krippendorff's alpha for the coding schemes was 0.866 and reached an acceptable level.

## Results

To answer the research questions, we performed *t*-tests, one-way analyses of variance (ANOVA) and multivariate analyses of variance (MANOVA) using a bootstrap method (*N* = 1,000), which are described in detail in the respective sections. All statistical analyses were performed using IBM SPSS 26.

### Overall Message Frames Usage

RQ1 addressed the use of five frames and promotion of health knowledge in opinion leader's microblog posts regarding physician–patient incidents. Overall, the conflict frame (*n* = 372) was the most dominant one, followed by the negative frame (*n* = 320), attribution frame (*n* = 308), and positive frame (*n* = 305). The use of the cooperation frame (*n* = 103) and health knowledge promotion (*n* = 89) was significantly less (Table 1). According to paired samples *t*-tests, the conflict frame (*M* = 0.125) was used more than the cooperation frame (*M* = 0.043), *t*_(940)_ = 10.969, *p* < 0.001, while the difference between the negative frame (*M* = 0.230) and the positive frame (*M* = 0.231) was not significant (*p* = 0.971).

### Changes Before and After COVID-19

RQ2 aimed to identify the differences in the use of message frames before and after the COVID-19 pandemic. Considering the potential error caused by unequal sample size, we performed Welch's *t*-test to answer this question. Compared to the pre-pandemic posts, the post-pandemic ones used more cooperation frame (M _pre−pandemic_ = 0.020, M _post−pandemic_ = 0.129; Welch's *t* = −7.324, *p* < 0.001), positive frame (M _pre−pandemic_ = 0.153, M _post−pandemic_ = 0.521; Welch's *t* = −11.009, *p* < 0.001), and negative frame (M _pre−pandemic_ = 0.206, M _post−pandemic_ = 0.320; Welch's *t* = −3.385, *p* < 0.001). In contrast, pre-pandemic posts mentioned less health-related knowledge (M _pre−pandemic_ = 0.062, M _post−pandemic_ = 0.010; Welch's *t* = 5.820, *p* < 0.001). However, no significant difference was found in the use of conflict and attribution frames, Welch's *t* = 1.872, *p* = 0.062 and Welch's *t* = 1.948, *p* = 0.052, respectively (Table 2).

**Table 2:** Mean and standard deviations of the Weibo frames before/after the pandemic (*n* = 941).

	**Pre-pandemic (*n* = 742)**	**Post-pandemic (*n* = 199)**	**Welch's *t***	***p-*value**
Conflict	0.130 (0.184)	0.107 (0.147)	1.872	0.062
Cooperation	0.020 (0.098)	0.129 (0.205)	−7.324	<0.001
Attribution	0.140 (0.213)	0.109 (0.198)	1.948	0.052
Positive	0.153 (0.308)	0.521 (0.444)	−11.009	<0.001
Negative	0.206 (0.338)	0.320 (0.440)	−3.385	<0.001
Promotion of health knowledge	0.062 (0.177)	0.010 (0.086)	5.820	<0.001

*95% CI refers to 95% confidence interval*.

We further tested the use of the message frame by opinion leaders from different communities before and after the COVID-19 pandemic. The results indicated that, for the media opinion leaders, the use of cooperation and positive frames significantly increased after COVID-19; M _pre−pandemic_ = 0.032, M _post−pandemic_ = 0.153; Welch's *t* = −5.977, *p* < 0.001; and M _pre−pandemic_ = 0.251, M _post−pandemic_ = 0.664; Welch's *t* = −9.878, *p* < 0.001, respectively. In contrast, the use of conflict and attribution frames significantly decreased after COVID-19; M _pre−pandemic_ = 0.175, M _post−pandemic_ = 0.074; Welch's *t* = 6.423, *p* < 0.001; and M _pre−pandemic_ = 0.186, M _post−pandemic_ = 0.102; Welch's *t* = 3.953, *p* < 0.001, respectively. However, the use of negative frame and promotion of health knowledge was insignificant (Welch's *t* = 0.372, *p* = 0.710 and Welch's *t* = 1.051, *p* = 0.294, respectively).

Very similar results were found regarding the variations in the use of cooperation and positive frames and health knowledge promotion for the general public opinion leaders. The use of cooperation and positive frames significantly increased after COVID-19, and health knowledge promotion significantly decreased; M _pre−pandemic_ = 0.008, M _post−pandemic_ = 0.080; Welch's *t* = −2.260, *p* = 0.033; M _pre−pandemic_ = 0.062, M _post−pandemic_ = 0.387; Welch's *t* = −3.843, *p* < 0.001; and M _pre−pandemic_ = 0.112, M _post−pandemic_ = 0.020; *t*_(180)_ = 3.412, *p* = 0.001, respectively. However, no significant results were found regarding the use of conflict, attribution, and negative frames; Welch's *t* = 0.104, *p* = 0.917; Welch's *t* = 1.991, *p* = 0.052; and Welch's *t* = −1.391, *p* = 0.175, respectively.

For physician opinion leaders, the use of use of conflict (M _pre−pandemic_ = 0.107, M _post−pandemic_ = 0.193; Welch's *t* = −3.212, *p* = 0.002), cooperation (M _pre−pandemic_ = 0.013, M _post−pandemic_ = 0.099; Welch's *t* = −2.955, *p* = 0.005), positive (M _pre−pandemic_ = 0.094, M _post−pandemic_ = 0.258; Welch's *t* = −2.882) and negative frames (M _pre−pandemic_ = 0.230, M _post−pandemic_ = 0.648; Welch's *t* = −6.224, *p* < 0.001) significantly increased after COVID-19, whereas the promotion of health knowledge significantly decreased (M _pre−pandemic_ = 0.076, M _post−pandemic_ = 0.000; Welch's *t* = 6.499, *p* < 0.001). No significant results were found regarding the use of attribution, Welch's *t* = −1.449, *p* = 0.152 (Table 3).

**Table 3:** Mean and standard deviations of the Weibo frames before/after the pandemic among trilateral opinion leaders.

	**Pre-pandemic (n = 742)**	**Post-pandemic (n = 199)**	**Welch's *t***	***p-*value**
**Media**				
Conflict	0.175 (0.198)	0.074 (0.119)	6.423	<0.001
Cooperation	0.032 (0.121)	0.153 (0.210)	−5.977	<0.001
Attribution	0.186 (0.222)	0.102 (0.187)	3.953	<0.001
Positive	0.251 (0.382)	0.664 (0.407)	−9.878	<0.001
Negative	0.193 (0.335)	0.179 (0.358)	0.372	0.710
Popular medical science	0.024 (0.115)	0.012 (0.101)	1.051	0.294
**General public**				
Conflict	0.083 (0.156)	0.080 (0.119)	0.104	0.917
Cooperation	0.008 (0.072)	0.080 (0.157)	−2.260	0.033
Attribution	0.132 (0.226)	0.067 (0.136)	1.991	0.052
Positive	0.062 (0.185)	0.387 (0.416)	−3.843	<0.001
Negative	0.191 (0.316)	0.307 (0.396)	−1.391	0.175
Popular medical science	0.112 (0.224)	0.020 (0.100)	3.412	0.001
**Physician**				
Conflict	0.107 (0.172)	0.193 (0.181)	−3.212	0.002
Cooperation	0.013 (0.079)	0.099 (0.210)	−2.955	0.005
Attribution	0.094 (0.184)	0.145 (0.240)	−1.449	0.152
Positive	0.094 (0.229)	0.258 (0.401)	−2.882	0.006
Negative	0.230 (0.353)	0.648 (0.464)	−6.224	<0.001
Popular medical science	0.076 (0.194)	0.000 (0.000)	6.499	<0.001

### Differentiated Message Frame Use by Opinion Leaders From Different Communities

MANOVA was run to address RQ3 about the overall differences in message frames among the opinion leaders from the general public, physicians, and media (Table 4); a series of ANOVA were conducted to measure the specific differences for each code item (Table 1a); paired sample *t*-test was used to compare the use of valance frames among different types of microbloggers.

**Table 4:** Mean and standard deviations of the Weibo frames in different types of opinion leaders.

**Account type**	**Conflict**	**Cooperation**	**Attribution**	**Positive**	**Negative**	**Popular medical science**
Media (*n* = 430)	0.146 (0.185)^a^	0.066 (0.161)^a^	0.162 (0.216)^a^	0.367 (0.431)^a^	0.189 (0.342)^a^	0.021 (0.111)^a^
General public (*n* = 182)	0.082 (0.151)^b^	0.018 (0.091)^b^	0.123 (0.216)^ab^	0.107 (0.255)^b^	0.207 (0.329)^a^	0.099 (0.213)^b^
Physician (*n* = 329)	0.121 (0.176)^a^	0.027 (0.115)^b^	0.102 (0.195)^b^	0.121 (0.270)^b^	0.297 (0.403)^b^	0.064 (0.180)^c^
All posts (*n* = 941)	0.125 (0.177)	0.043 (0.136)	0.134 (0.210)	0.231 (0.372)	0.230 (0.365)	0.051 (0.163)
*F*_(2,938)_	8.672	11.785	7.925	60.105	8.715	16.684
*p*	<0.001	<0.001	<0.001	<0.001	<0.001	<0.001

a, b, c, *different superscripts indicate the existence of a statistically significant difference*.

The MANOVA results indicated an overall difference in post framing by microblogger's type: *F*_(12,1864)_ = 16.287, *p* < 0.001; Wilk's Λ = 0.819; partial η^2^ = 0.095.

The *post-hoc* tests using Tukey's HSD revealed that media's use of the conflict frame (*M* = 0.146) was significantly higher than that of the general public opinion leaders (*M* = 0.082), *p* < 0.001; however, no significant difference was identified between the media and the physicians (*M* = 0.121, *p* = 0.114) and between the general public and the physicians (*p* = 0.052). Item-specific tests suggested that the media opinion leaders concentrated more on physician-patient conflict (M _media_ = 0.34) in their posts than the general public (M _public_ = 0.09, *p* < 0.001) and the physician opinion leaders (M _physician_= 0.25, *p* = 0.007). Similarity, the overall use of cooperation frame was significantly higher by media opinion leaders (M _media_ = 0.066) than that of the general public opinion leaders (M _public_ = 0.018, *p* < 0.001) and the physician opinion leaders (M _physician_ = 0.027, *p* < 0.001), but the difference between general public and physicians opinion leaders was insignificant (*p* = 0.767). The following item-specific tests showed that media opinion leaders emphasized general cooperation (M _media_ = 0.14, M _public_ = 0.03, *p* < 0.001), physician-patient communication (M _media_ = 0.06, M _public_ = 0.02, *p* = 0.050), and physician's understanding (M _media_ = 0.04, M _public_ = 0.00, *p* = 0.044) more than general public opinion leaders. In general, the media use attribution frame significantly higher than that of the physician opinion leaders (M _media_ = 0.162, M _physician_ = 0.102, *p* < 0.001), but the difference between the opinion leaders from the general public and physicians was insignificant (*p* = 0.580); following item-specific tests found that media tend to attribute the responsibility to governmental (M _media_ = 0.27, M _physician_ = 0.17, *p* = 0.004) and physician (M _media_ = 0.11, M _physician_ = 0.06, *p* = 0.020) than the physician opinion leaders.

Regarding the use of valance frames, the media use positive frame significantly higher than that of the general public (M _media_ = 0.367, M _public_ = 0.107, *p* < 0.001) and the physician opinion leaders (M _physician_ = 0.121, *p* < 0.001); but the difference between the opinion leaders from the general public and physicians was insignificant (*p* = 0.906). Regarding the use of the negative frame, the physicians' use (*M* = 0.297) was significantly higher than that of both the media (M = 0.189; *p* < 0.001) and general public (*M* = 0.207; *p* = 0.019); the difference between the media and the general public was insignificant (*p* = 0.855). Regarding health knowledge promotion, media (*M* = 0.021) was less likely to mention health knowledge than the general public (M = 0.099; *p* < 0.001) and physicians (*M* = 0.064, *p* = 0.001), and the physicians mentioned it less than the general public (*p* = 0.044). Paired sample *t*-test indicated that media opinion leaders used more positive than negative frames (M _positive_ = 0.431, M _negative_ = 0.342, *p* < 0.001), while general public (M _positive_ = 0.255, M _negative_ = 0.329, *p* = 0.002) and physician opinion leaders (M _positive_ = 0.270, M _negative_ = 0.403, *p* < 0.001) used more negative than positive frames.

### Frames Used in Different Types of Incidents

RQ4 addresses whether the use of five frames and the promotion of health knowledge in the posts differed by the type of physician–patient incidents. MANOVA was used to test the overall differences (Table 5), and series of ANOVA were conducted to measure the specific differences for each code item (Table 1b), and a paired sample *t*-test was used to compare the differences in the use of valance frames for specific physician-patient incidents. The MANOVA results suggested an overall statistically significant difference in the framing of the posts based on incident type: *F*_(12,1864)_ = 17.334, *p* < 0.001; Wilk's Λ = 0.809; partial η^2^ = 0.100.

**Table 5:** Mean and standard deviations of the Weibo frames in different types of physician-patient incident.

**Incident type**	**Conflict**	**Cooperation**	**Attribution**	**Positive**	**Negative**	**Popular medical science**
Incidents of violence against physicians (*n* = 661)	0.126 (0.163)^a^	0.056 (0.155)^a^	0.125 (0.204)^a^	0.299 (0.414)^a^	0.261 (0.399)^a^	0.036 (0.146)^a^
No-death incident (*n* = 67)	0.090 (0.142)^a^	0.037 (0.100)^ab^	0.070 (0.148)^a^	0.110 (0.187)^b^	0.045 (0.141)^b^	0.097 (0.199)^b^
Patient-death incident (*n* = 213)	0.133 (0.223)^a^	0.005 (0.048)^b^	0.182 (0.237)^b^	0.060 (0.150)^b^	0.193 (0.271)^c^	0.082 (0.192)^b^
All posts (*n* = 941)	0.125 (0.177)	0.043 (0.136)	0.134 (0.210)	0.231 (0.372)	0.230 (0.365)	0.051 (0.163)
*F*_(2,938)_	1.561	11.628	9.412	40.076	12.467	9.383
*p-*value	0.210	<0.001	<0.001	<0.001	<0.001	<0.001

a, b, c, *different superscripts indicate the existence of a statistically significant difference*.

The MANOVA results indicated that the overall use of conflict frame did not differ by the type of incident (patient death *vs*. incidents of violence against physicians, *p* = 0.899; patient death incidents *vs*. no death incidents, *p* = 0.192; and incidents of violence against physicians *vs*. no death incidents, *p* = 0.234). However, item-specific tests suggested that violence against physician incidents stressed more physician-patient conflict (M _physician_ = 0.33, M _patient_ = 0.14, *p* < 0.001), less patient-public disagreement (M _physician_ = 0.01, M _patient_ = 0.08, *p* < 0.001) and less physician-public disagreement (M _physician_ = 0.04, M _patient_ = 0.10, *p* = 0.002) than patient-death incidents. The overall use of cooperation frame in the posts regarding incidents of violence against physicians (M = 0.056) was significantly more than that of patient death incidents (*M* = 0.005; *p* < 0.001); neither the difference between incidents of violence against physicians and no death incidents (*M* = 0.037, *p* = 0.535) nor the difference between patient death incidents and no death incidents (*p* = 0.193) was significant; item-specific tests suggested that violence against physician incidents emphasized general cooperation (M _physician_ = 0.11, M _patient_ = 0.01, *p* < 0.001), physician-patient communication (M _physician_ = 0.05, M _patient_ = 0.01, *p* = 0.027), physician's understanding (M _physician_ = 0.04, M _patient_ = 0.00, *p* = 0.008) and patient's understanding (M _physician_ = 0.03, M _patient_ = 0.00, *p* = 0.042) more than patient-death incidents. Overall speaking, the attribution frame was used more when discussing patient death incidents (*M* = 0.182) than incidents of violence against physicians (*M* = 0.125) and no death incidents (*M* = 0.070) at *p* < 0.001, but no difference was found between incidents of violence against physicians and no death incidents (*p* = 0.103); following item-specific tests suggested that violence against physician incidents emphasized less physician attribution (M _physician_ = 0.03, M _patient_ = 0.24, *p* < 0.001) and more patient attribution (M _physician_ = 0.12, M _patient_ = 0.06, *p* = 0.049) than patient-death incidents.

The positive frame was used more when discussing incidents of violence against physicians (*M* = 0.299) than patient death incidents (*M* = 0.060) and no death incidents (*M* = 0.070) at *p* < 0.001; the difference between patient death incidents and no death incidents was insignificant (*p* = 0.579). Likewise, the negative frame was used more in violence against physicians (*M* = 0.261) than patient death incidents (*M* = 0.193; *p* = 0.042) and no death incidents (*M* = 0.045; *p* < 0.001), and in posts discussing patient death incidents than no death incidents (*p* = 0.010). Paired sample *t*-test showed that no death incidents used more positive than negative frames (*p* = 0.033), patient death incidents used more negative than positive frames (*p* = 0.001), and there was no significant difference in the use of valance frames in incidents of violence against physicians (*p* = 0.169).The promotion of health knowledge was significantly less in incidents of violence against physicians (*M* = 0.036) than patient death incidents (*M* = 0.082; *p* = 0.001) and no death incidents (*M* = 0.097; *p* = 0.010), and the difference between patient death incidents and no death incidents was insignificant (*p* = 0.790).

## Discussion

Opinion leaders on social media engaged in constructing and influencing public's understanding these controversial incidents through utilizing different post frames. The microblog post from opinion leaders on physician-patient conflicts have become objective history texts, which enable us to explore the opinions, interplay and change of different communities on physician-patient conflict incidents. This study content analyzed the microblog post frames of media, general public and health professions opinion leaders on physician-patient conflicts in the past ten years. Through comparing the message frames among different groups, and exploring the changes in the frame of the posts before and after the COVID-19 pandemic, findings shed light on the underlying norms, interest and value propositions held by different groups. It is an important part of public opinion of physician–patient relationship (44), and also creates an objective empirical structure for further exploring physician–patient and other related health communications *via* social media.

The results indicated that the media opinion leaders used systematically biased framing of physician–patient conflicts. Among the three groups of opinion leaders, the media use more conflict frames while making less effort to promote health knowledge than the general public and physician opinion leaders. Specifically, media concentrated on physician-patient conflicts, while physician opinion leaders more focused on the disagreement between physician and public. This difference indicated that media intend to capture public's attention through portraying conflicts while physicians aimed to clarify the incidents.

The average followers of media (mean = 62,420,699) are several times those of the general public (mean = 9,891,605) and physician (mean = 2,540,837) microbloggers; hence, the media probably has a greater influence on public opinion. Media microbloggers concentrate on depicting physician–patient conflicts rather than promoting incident-related health knowledge; this type of deviation is misleading and biases the public perception, thus hurting physician–patient trust and relationship, creating encounter difficulties, and causing a vicious circle of physician–patient communication (2).

Media opinion leaders used more positive frame than negative frame. Since Chinese media are mostly stated-controlled, they tend to shape public perception of harmonious society through using positive message frame. On the other hand, general public and physician used more negative than positive frames. The significantly high use of negative frames by physician opinion leaders reflect that health profession's feelings are hurt by intense physician- patient conflicts, which will inevitably cause physician to be more cautious and self-protection even in the face-to-face communication.

It is noteworthy that negative and positive frames were more used, while attribution frame was less used in violence against physician than the other two types of conflicts. The high utilization of valence (negative/positive) frames reflect great concern and strong sentiment on violence against physician incidents. However, less utilization of attribution frames may lead to fewer reflection of the social and systematical causes of the series of malicious attack on physicians.

The general public and physician opinion leaders shared something in common: they mostly attributed the cause of conflicts to the government/society (22.7% general public and 16.7% physician), while attributing the least to the patient (7.1% general public and 8.5% physician), which indicated that both of these two groups realized the health system and limit medical care resource are main causes for physician-patient conflicts. Moreover, there are no significant differences in the use of conflict, cooperation, negative, and popular science frames, implying that no fundamental discrepancy exists between the general public and health professionals regarding physician–patient conflict incidents.

Although the tensions between physicians and patients in China have some special reasons, such as conflicts between the financial interests of health institutions and patient's appropriate treatment, and contradictions among a large number of patients and limited medical resources (45), physicians and the general public still share many common views on physician–patient conflict incidents. By expressing and viewing other's opinions on health issues, health professionals and the general public could further promote mutual understanding, enhance public health education, and strengthen physician–patient communication, thus improving the physician–patient relationship.

After the pandemic, Chinese government has highlighted the praise of physicians and actively guided public opinion in the hope of building a more harmonious physician–patient relationship, while the selfless dedication shown by healthcare workers during the pandemic made the general public more understanding and sympathetic to physicians (46). The findings of the study corroborated these changing tends. In general, more positive, negative and cooperation frames are being utilized to construct posts of physician–patient conflicts after the COVID-19 pandemic. Specifically, media microbloggers used more positive and cooperation frames, while using less conflict and attribution frames on physician–patient incidents after the COVID-19 pandemic. This contributes to improving physician–patient relationships since negative media portrayal of physicians led to physician–patient tension (47). The public also shows more understanding and gratitude to health professionals during the COVID-19 pandemic (48). Therefore, there is an improving trend of physician–patient relationship in China, while physician–patient relationship has become intense in some other countries due to social distancing and limited diagnostic time (49, 50). Future research could further explore the changes in physician–patient communication and trust, and their influence on physician–patient relationship and patient adherence after the COVID-19 pandemic.

Although this study did not directly investigate the public's understanding and behavior to different post frames, previous studies have provided ample evidence of the significant relationship between public reactions and message frames (51, 52). Findings of this study provide empirical data structure for physician-patient communication on social media. Further efforts should be made to set up and enhance communication between health professionals and the general public on social media, since previous studies showed that Internet usage aggravated mistrust between physicians and patients in China (53). Moreover, because the media opinion leaders have a greater number of followers than the general public and physician opinion leaders on microblog, it is essential to encourage media microbloggers to make efforts to popularize medical science and use balanced news frames on health issues to enhance public health education and improve physician–patient mutual understanding and relationship.

## Limitations

This study has several limitations. First, the study was conducted in China, which may limit the generalizability of the findings, especially in Western countries with different cultural backgrounds and medical systems. Future studies could further explore how to utilize online opinion leaders to promote health communication in different contexts. Second, this study did not analyse the retweets and comments of microblog posts. Future studies should further analyse the contents of the comments and retweets of popular microblog posts to analyse the follower's reactions to the health opinion leaders. Furthermore, the completeness of the collected posts may be open to questions. It is highly likely that some influential posts were removed before the data were collected because this study involved some sensitive physician–patient incidents, such as death-related incidents, and some incidents were not the most recent. In addition, unequal sample sizes may reduce the contribution of the results, and we can only eliminate potential negative effects at the statistical level. All these conditions increase the challenges involved in accessing all the posts on each incident.

## Conclusions

This study conducted a content analysis to examine how opinion leaders from the physicians, general public, and media on microblog framed posts regarding major physician–patient conflicts. The media use significantly more conflict and attribution frames and devote the least effort to promote health knowledge. This imbalanced use of news frames would cultivate and reinforce the public perception of physician–patient contradictions. More cooperation and positive frames were used after the COVID-19 pandemic, indicating an improvement in the physician–patient relationship in China.

## Data Availability Statement

The raw data supporting the conclusions of this article will be made available by the authors, without undue reservation.

## Author Contributions

WG and QG: designed the study, methodology, writing—original draft preparation, and writing—review and editing. QG: software and data curation. WG: supervision, project administration, funding acquisition, conceptualization, and resources. Both authors have read and agreed to the published version of the manuscript.

## Funding

The paper is supported by grants from National Natural Science Foundation of China (No. 71802058), MOE (Ministry of Education in China) Project of Humanities and Social Sciences (No. 18YJC860007), Natural Science Foundation of Guangdong Province (No. 2018A0303100008), and the National Social Science Foundation of China (No.18BXW062).

## Conflict of Interest

The authors declare that the research was conducted in the absence of any commercial or financial relationships that could be construed as a potential conflict of interest.

## Publisher's Note

All claims expressed in this article are solely those of the authors and do not necessarily represent those of their affiliated organizations, or those of the publisher, the editors and the reviewers. Any product that may be evaluated in this article, or claim that may be made by its manufacturer, is not guaranteed or endorsed by the publisher.

## References

[1] Chinese Medical Health System Reform Report. The Number of Medical Disputes in China Has Increased 10-Fold in the Past 10 Years. Available online at: https://www.medsci.cn/article/show_article.do;jsessionid=E189CC96EDBD4489C91F5CA4A9B59414?id=ac1162312a9 (accessed December 10, 2018).

[2] Nie JB Cheng Y Zou X Gong N Tucker JD Wong B. The vicious circle of patient-physician mistrust in China: health professionals' perspectives, institutional conflict of interest, and building trust through medical professionalism. Dev World Bioeth. (2018) 18:26–36. 10.1111/dewb.12170 28922547

[3] Zhong ZJ. How the doctor-patient relationship affects the patients' following of doctor's orders: from an interpersonal communication perspective. Acad Res. (2018) 4:67–73. Available online at: https://kns.cnki.net/kcms/detail/detail.aspx?dbcode=CJFD&dbname=CJFDLAST2018&filename=XSYJ201804010&uniplatform=NZKPT&v=c3BgPzeYa4oDFn-Yv1xuMYmNGCa5fOW2F1xB–wzEtZHAkLM4b0uk95Fmvt47RoI

[4] Feng J Li Y Han C Xu L Duan L. A retrospective analysis on 418 medical disputes (in Chinese). Chinese Hosp Manag. (2013) 33:77–9. Available online at: https://kns.cnki.net/kcms/detail/detail.aspx?dbcode=CJFD&dbname=CJFDHIS2&filename=YYGL201309046&uniplatform=NZKPT&v=ml6c1xLKeZrCJyP5UCfZOW6_dYWOETo1jWawPfv2gqPGGj0cd3vXMBEBilAbWlvF

[5] Alpert JM Womble FE. Just What the doctor tweeted: physicians' challenges and rewards of using twitter. Health Commun. (2016) 31:824–32. 10.1080/10410236.2015.1007551 26644165

[6] Smailhodzic E Hooijsma W Boonstra A Langley DJ. Social media use in healthcare: a systematic review of effects on patients and on their relationship with healthcare professionals. BMC Health Serv Res. (2016) 16:442. 10.1186/s12913-016-1691-0 27562728 PMC5000484

[7] Lu Y Wu Y Liu J Li J Zhang P. Understanding health care social media use from different stakeholder perspectives: a content analysis of an online health community. J Med Internet Res. (2017) 19:e109. 10.2196/jmir.7087 28389418 PMC5400888

[8] Kreps GL Neuhauser L. New directions in eHealth communication: opportunities and challenges. Patient Educ Couns. (2010) 78:329–36. 10.1016/j.pec.2010.01.013 20202779

[9] Wu T Deng Z Feng Z Gaskin DJ Zhang D Wang R. The effect of doctor-consumer interaction on social media on consumers' health behaviors: cross-sectional study. J Med Internet Res. (2018) 20:e73. 10.2196/jmir.9003 29490892 PMC5852273

[10] Katz E. The two-step flow of communication: an up-to-date report on a hypothesis. Public Opin Q. (1957) 21:61–78. 10.1086/266687

[11] Valente TW Pumpuang P. Identifying opinion leaders to promote behavior change. Health Educ Behav. (2017) 34:881–96. 10.1177/1090198106297855 17602096

[12] Hilbert M Vasquez J Halpern D Valenzuela S Arriagada E. One step, two step, network step? complementary perspectives on communication flows in twittered citizen protests. Soc Sci Comput Rev. (2017) 35:444–61. 10.1177/0894439316639561

[13] Han GK Wang W. Mapping user relationships for health information diffusion on microblogging in china: a social network analysis of sina weibo. Asian J Commun. (2015) 25:65–83. 10.1080/01292986.2014.989239

[14] Korda H Itani Z. Harnessing social media for health promotion and behavior change. Health Promot Pract. (2013) 14:15–23. 10.1177/1524839911405850 21558472

[15] Chu K-H Majmundar A Allem J-P Soto DW Cruz TB Unger JB. Tobacco use behaviors, attitudes, and demographic characteristics of tobacco opinion leaders and their followers: twitter analysis. J Med Internet Res. (2019) 21:e12676. 10.2196/12676 31165716 PMC6746100

[16] Griner SB Thompson EL Vamos CA Chaturvedi AK Vazquez-Otero C Merrell LK. Dental opinion leaders' perspectives on barriers and facilitators to HPV-related prevention. Hum Vaccin Immunother. (2019) 15:1856–62. 10.1080/21645515.2019.1565261 30735476 PMC6746486

[17] Hu G Han X Zhou H Liu Y. Public perception on healthcare services: evidence from social media platforms in China. Int J Environ Res Public Health. (2019) 16:1273. 10.3390/ijerph16071273 30974729 PMC6479867

[18] Chen H Gao Y. Restructure of discourse power in doctor-patient relationship. J Commun. (2013) 11:68–89. Available online at: https://kns.cnki.net/kcms/detail/detail.aspx?dbcode=CJFD&dbname=CJFDHIS2&filename=YANJ201311009&uniplatform=NZKPT&v=UU9SauABo0yvRYNUsO-h2cnoYgT5QlOZvJ7sX_3TvBBiBsXQtv5WaAkfTvLYVuse

[19] Entman RM. Framing: toward clarification of a fractured paradigm. J Commun. (1993) 43:51–8. 10.1111/j.1460-2466.1993.tb01304.x

[20] Nip JYM Fu K. Networked framing between source posts and their reposts: an analysis of public opinion on China's microblogs. Inf Commun Soc. (2016) 19:1127–49. 10.1080/1369118X.2015.1104372

[21] Wasike BS. Framing news in 140 characters: How social media editors frame the news and interact with audiences via twitter. Glob Media J Can. (2013) 6:5–23.

[22] Duan G Liao X Yu W Li G. Classification and prediction of violence against Chinese medical staff on the Sina microblog based on a self-organizing map: quantitative study. J Med internet Res. (2020) 22:e13294. 10.2196/13294 32348253 PMC7284412

[23] Gamson WA Modigliani A. Media discourse and public opinion on nuclear power: a constructionist approach. Am J Sociol. (1989) 95:1–37. 10.1086/229213

[24] Protecting Chinese doctors. Lancet. (2020) 395:90. 10.1016/S0140-6736(20)30003-9 31929017

[25] Gao B Dong J. Does the impact of COVID-19 improve the doctor-patient relationship in China? Am J Med Sci. (2020) 360:305. 10.1016/j.amjms.2020.05.039 32563570 PMC7831870

[26] Nie JB Li L Gillet G Tucker JD Kleinman A. The crisis of patient-physician trust and bioethics: lessons and inspirations from China. Dev World Bioeth. (2018) 18:56–64. 10.1111/dewb.12169 28922581

[27] Bauhoff S. Systematic self-report bias in health data: impact on estimating cross-sectional and treatment effects. Heal Serv Outcomes Res Methodol. (2011) 11:44–53. 10.1007/s10742-011-0069-3

[28] Althubaiti A. Information bias in health research: definition, pitfalls, and adjustment methods. J Multidiscip Healthc. (2016) 9:211–7. 10.2147/JMDH.S104807 27217764 PMC4862344

[29] Matthes J. What's in a frame? a content analysis of media framing studies in the world's leading communication journals, 1990-2005. Journal Mass Commun Q. (2009) 86:349-67. 10.1177/107769900908600206

[30] Burscher B Odijk D Vliegenthart R Rijke Mde Vreese CHde. Teaching the computer to code frames in news: comparing two supervised machine learning approaches to frame analysis. Commun Methods Meas. (2014) 8:190–206. 10.1080/19312458.2014.937527

[31] Semetko H Valkenburg P. Framing European politics: a content analysis of press and television news. J Commun. (2000) 50:93–109. 10.1111/j.1460-2466.2000.tb02843.x

[32] Gong W Tu C Jiang LC. Stigmatized portrayals of single women: a content analysis of news coverage on single women and single men in China. J Gend Stud. (2017) 26:197–211. 10.1080/09589236.2015.1095082

[33] Gross K. Framing persuasive appeals: episodic and thematic framing, emotional response, and policy opinion. Polit Psychol. (2008) 29:169–92. 10.1111/j.1467-9221.2008.00622.x

[34] Schuck ART de Vreese CH. Between risk and opportunity. Eur J Commun. (2006) 21:5–32. 10.1177/0267323106060987

[35] Nisar MT Prabhakarb G. Trains and twitter: firm generated content, consumer relationship management and message framing. Transp Res Part A Policy Pract. (2018) 113:318–34. 10.1016/j.tra.2018.04.026

[36] Shi J Salmon CT. Identifying opinion leaders to promote organ donation on social media: network study. J Med Internet Res. (2018) 20:e7. 10.2196/jmir.7643 29317384 PMC5780616

[37] Sina Commands 56% of China's Microblog Market. Resonance. Available online at: http://www.resonancechina.com/sina-commands-56-of-chinas-microblog-market/ (accessed September 13, 2019).

[38] Liao H Wang Y Guan P. Topic mining and viewpoint recognition of different communicators in the transmission cycle of micro-blog public opinion. Libr Inf Serv. (2018) 62:77. 10.13266/j.issn.0252-3116.2018.19.010

[39] Ji P. Emotional criticism as public engagement: how weibo users discuss “peking university statues wear face-masks”. Telemat Informatics. (2016) 33:514-24. 10.1016/j.tele.2015.06.017

[40] Marcus GE Mackuen MB. Anxiety, enthusiasm, and the vote: the emotional underpinnings of learning and involvement during presidential campaigns. Am Polit Sci Rev. (1993) 87:672–85. 10.2307/2938743

[41] Valenzuela S Piña M Ramírez J. Behavioral effects of framing on social media users: How conflict, economic, human interest, and morality frames drive news sharing. J Commun. (2017) 67:803–26. 10.1111/jcom.12325

[42] Zhao S. Research on the accurate design for promoting mainstream discourse guidance from the perspective of internet public opinion field (in Chinese). J Sichuan Univ. (2020) 3:12–9. Available online at: https://kns.cnki.net/kcms/detail/detail.aspx?dbcode=CJFD&dbname=CJFDLAST2020&filename=SCDZ202003003&uniplatform=NZKPT&v=C16la8CDkaiqRTnBpoV-D8EzsefoIYFFyUYWfhmWVHXkcBauvktzLqJGJV_AE4J

[43] Theall KP Fleckman J Jacobs M. Impact of a community popular opinion leader intervention among African American adults in a southeastern United States community. AIDS Educ Prev. (2015) 27:275–87. 10.1521/aeap.2015.27.3.275 26010317 PMC4612363

[44] Bosslet GT Torke AM Hickman SE Terry CL Helft PR. The patient-doctor relationship and online social networks: results of a national survey. J Gen Intern Med. (2011) 26:1168–74. 10.1007/s11606-011-1761-2 21706268 PMC3181288

[45] Zhong ZJ Nie J Xie X Liu K. How medic–patient communication and relationship influence Chinese patients' treatment adherence. J Health Commun. (2019) 24:29–37. 10.1080/10810730.2018.1561768 30596351

[46] Liu JH. Building a new type of doctor-patient relationship to empower the construction of health China. People Tribune. (2021) 33:86–8. Available online at: https://kns.cnki.net/kcms/detail/detail.aspx?dbcode=CJFD&dbname=CJFDLAST2021&filename=RMLT202133021&uniplatform=NZKPT&v=lL6ADhrA_19CCh2Fw8UtrwLCRvEnsXgNJt3sSXIYitWDm5LRsh1zEBklNz_LCF_N

[47] Meng H. Understanding professional medical trouble makers. Chin Med Guide. (2006) 8:15–6. 10.15912/j.cnki.gocm.2006.08.005

[48] Liu Q Luo D Haase JE Guo Q Wang XQ Liu S. The experiences of health-care providers during the COVID-19 crisis in China: a qualitative study. Lancet Glob Health. (2020) 8:e790–8. 10.1016/S2214-109X(20)30204-7 32573443 PMC7190296

[49] Nwoga HO Ajuba MO Ezeoke UE. Effect of COVID-19 on doctor-patient relationship. Int J Commun Med Public Health. (2020) 7:4690. 10.18203/2394-6040.ijcmph20205136

[50] Assegaff S Asri SDA Rinaldi EA Sutopo H Dhani R. Stigmatization, Dishonest Patients, and Challenges of Diagnosing COVID-19: A Review of Physician-Patient Communication in Indonesia. Available online at: 10.2139/ssrn.3830425 (accessed March 10, 2021).

[51] Nabi RL Gustafson A Jensen R. Framing climate change: exploring the role of emotion in generating advocacy behavior. Sci Commun. (2018) 40:442–68. 10.1177/1075547018776019

[52] Parmer J Baur C Eroglu D Lubell K Prue C Reynolds B. Crisis and emergency risk messaging in mass media news stories: is the public getting the information they need to protect their health? Health Commun. (2016) 10:1215–22. 10.1080/10410236.2015.1049728 26940247

[53] Zhu BW Luo JJ. Does internet use affect public trust in doctors?– empirical analysis based on data CSS2013. Jiangsu soc sci. (2017) 3:70–8. 10.13858/j.cnki.cn32-1312/c.2017.03.01131878145

