# Supplementary material for: Framing Public Opinion on Physician-Patient Conflicts on Microblog: A Comparative Content Analysis
Source: Front Public Health. 2022 Feb 4;10:831638. doi: 10.3389/fpubh.2022.831638 (PMC8854764; doi:10.3389/fpubh.2022.831638)
Supplement: Supplementary file 1 [file Table_1.DOCX]

| Appendix 1. The description of selected patient–physician incidents. | | | | | | |
| --- | --- | --- | --- | --- | --- | --- |
| Type | Year | Incidents | Description | Time | Sources | Search words |
| Incidents of violence against physicians | 2012 | The fatal attack in Harbin Hospital (n = 137) | On Mar. 23, 2012, a patient aged 17 murdered a medical intern, aged 27, and attacked three medical staffs in Harbin Medical University Affiliated 1st Hospital. Upon examination, it was found that the murderer purposely attacked the physicians because he misunderstood the treatment offered by his doctor in charge. On Oct. 19, the murderer was sentenced to life imprisonment for his crimes. | 2012.3.23–2012. 3.30;  2012.10.19–2012.10.25 (trial) | GooSeeker | Harbin Hospital, fatal attacks against physician |
|  | 2013 | The fatal attack in Wen Ling Hospital (n = 128) | On Oct. 25, 2013, a patient murdered a physician and attacked 2 physicians in the First People's Hospital of Wen Ling. The murderer asserted that he killed the physicians because he believed the physicians misdiagnosed him and gave him the wrong treatment. On April. 1, 2014, the murderer was condemned to death. | 2013.10.25–2013.11.3;  2014.4.1–2014.4.7 (trial) | GooSeeker | Wen Ling Hospital, fatal attacks against physician; Wang Yunjie (physician’s name) |
|  | 2018 | The Peking University Hospital incident (n = 77) | On Sep. 22, 2018, an obstetrician and gynecologist was assaulted by a patient's family in Peking University First Hospital. | 2018.9.22–2018.9.28 | GooSeeker | Peking University Hospital, injury case, physician |
|  | 2019 | The Civil Aviation General Hospital incident (n = 120) | On Dec. 24, 2019, a killing of a doctor occurred in the emergency resuscitation room of Civil Aviation General Hospital in Chaoyang District, Beijing. Sun Wenbin, a patient's family member, slashed female doctor Yang Wen's neck with a knife, eventually killing Dr. Yang Wen. | 2019.12.24–2019.12.30 | GooSeeker | Civil Aviation General Hospital, fatal attacks, Yang Wen, Sun Wenbin |
|  | 2020-1 | The Beijing Chao-yang Hospital incident (n = 172) | On Jan. 20, 2020, Cui Zhenguo entered the outpatient building of Chaoyang Hospital with a pre-prepared kitchen knife and hacked Dr. Tao Yong in the back of the head, neck and arms, and successively wounded three other people who were blocking his attack. | 2020.1.20–2020.1.26 | GooSeeker | Beijing Chao-yang Hospital, attacks, Tao Yong, Cui Zhenguo |
|  | 2020-2 | The Yanqing Hospital incident (n = 27) | On April 29, 2020, Yang took his pregnant wife to the Yanqing Hospital for maternity checkup, and then hurt the physician because he was dissatisfied with the physician’s request. | 2020.4.29–2020.5.5 | GooSeeker | Yanqing Hospital, attacks, physician |
| No-death incidents | 2014 | Physicians took selfies in the operating room (n = 55) | Physicians took a selfie with the patient in the operating room after surgery. Physicians said that they took the picture to in memory of the old operating room and they got the patient's permission first. Despite that, administrative sanctions were imposed to punish such behaviors. | 2014.12.20–2014.12.26 | GooSeeker | Selfies in the operating room |
|  | 2015 | Physician fainted in the operating room (n = 12) | A physician from the People's Hospital of Jing Jiang, Jiangsu Province, suffered a sudden pain in the heart during surgery. He was given a painkiller and insisted on finish the surgery save the patient's life. After finishing the surgery, he fainted in the operating room. He was being rescued for 12 hours and finally awakened from his coma three days after. | 2015.7.16–2015.7.27 | GooSeeker | Physician suffered from sudden Disease; Hu Fangbin (physician’s name) |
| Patient-death incidents | 2016 | Wei Zexi incident (n = 142) | A 21-year old Chinese college student named Wei Zexi was diagnosed with synovial sarcoma, a rare form of cancer. He and his family learned from a promoted result on Baidu, a Chinese search engine, that the Second Hospital of the Beijing Armed Police Corps can provide appropriate treatment to this disease. However, such treatment proved unsuccessful and the medical information promoted by Baidu was turned out to be a fake information. Wei spend more than 200,000 Yuan in the hospital and died on April 12, 2016. Before his death, he shared his experience online and accused Baidu of promoting fake medical information. | 2016.5.1–2016.5.17 | Zhiweidata | Wei Zexi |
|  | 2017 | Yu Lin mother incident (n = 71) | On Aug. 31, 2017, a mother, named Ma Rongrong, jumped to her death from the 5th floor of the building of the First Hospital of Yu Lin. After investigation, it is found that the puerpera choice suicide because she cannot bear extreme labor pain. The hospital declared that they recommend the puerpera of cesarean delivery for three times, but all rejected by Ma's family. On the contrary, Ma's family insisted that it was the doctors who rejected their requirement of cesarean. | 2017.9.5–2017.9.18 | Zhiweidata | Yuling mother |

Appendix 2. Data collection procedure.


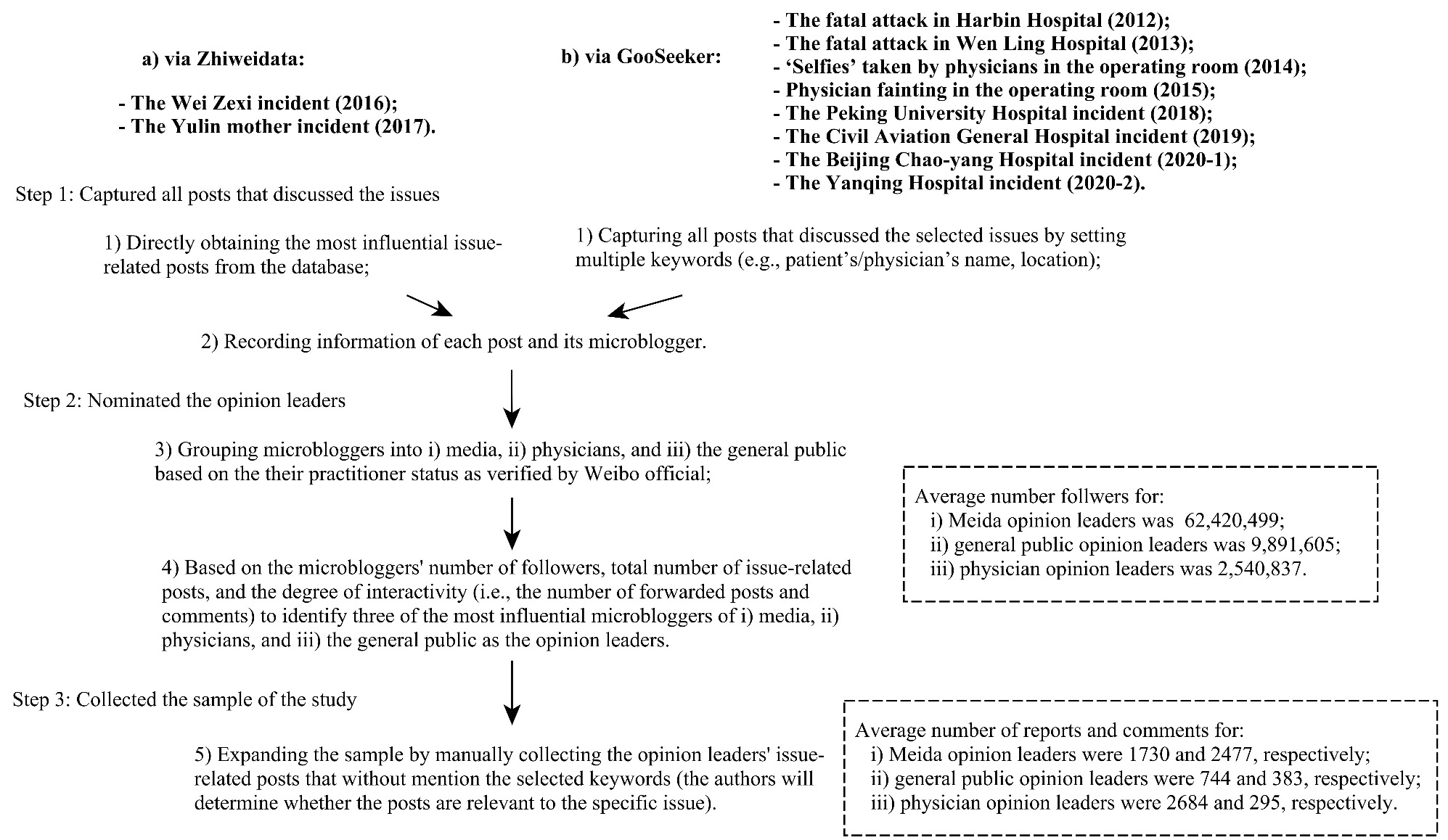


| Table 1a. Overall landscape of the frames uses by opinion leader types (bootstrapping *n* = 2000) | | | | | | |
| --- | --- | --- | --- | --- | --- | --- |
| Category | Coding description | Media | Public | Physician | *F* | *p* |
|  |  | n (mean) | n (mean) | n (mean) | *df*=2 |  |
| **Conflict** |  |  |  |  |  |  |
| Physician-patient conflict | Disagreement between patient/patient's family and doctor | 147 (0.34)^a^ | 17 (0.09)^b^ | 81 (0.25)^c^ | 21.648 | <0.001 |
| Patient-public disagreement | Disagreement between patient and the public opinion | 11 (0.03)^a^ | 4 (0.02)^a^ | 9 (0.03)^a^ | 0.068 | 0.934 |
| Physician-public disagreement | Disagreement between doctor and the public opinion | 17 (0.04)^a^ | 18 (0.10)^b^ | 24 (0.07)^ab^ | 4.312 | 0.014 |
| General conflict | Disagreement of two sides or to more than two sides of the problem or issue | 77 (0.18)^a^ | 21 (0.12)^a^ | 45 (0.14)^a^ | 2.470 | 0.085 |
|  |  | (0.146) | (0.082) | (0.121) | Mean=0.125 | |
| **Cooperation** |  |  |  |  |  |  |
| Cooperation | Cooperation between patient and doctor | 58 (0.14)^a^ | 5 (0.03)^b^ | 15 (0.05)^b^ | 14.735 | <0.001 |
| Communication | Good communication between patient/ patient's family and doctor | 24 (0.06)^a^ | 3 (0.02)^b^ | 9 (0.03)^ab^ | 3.520 | 0.030 |
| Physician's understanding | Patient’s concerns are well understood by the doctor | 17 (0.04)^a^ | 1 (0.00)^b^ | 7 (0.02)^ab^ | 3.150 | 0.043 |
| Patient's understanding | Doctors’ views are well understood by the patient | 14 (0.03)^a^ | 4 (0.02)^a^ | 4 (0.01)^a^ | 1.790 | 0.182 |
|  |  | (0.066) | (0.018) | (0.027) | Mean=0.043 | |
| **Attribution** |  |  |  |  |  |  |
| Government | Society/government has the responsibility to solve the problem | 114 (0.27)^a^ | 40 (0.22)^ab^ | 55 (0.17)^b^ | 5.219 | 0.006 |
| Physician | Doctors/hospital have the responsibility to solve the problem | 47 (0.11)^a^ | 14 (0.08)^ab^ | 18 (0.06)^b^ | 3.701 | 0.025 |
| Patient | Patient or family has the responsibility to solve the problem | 48 (0.11)^a^ | 13 (0.07)^a^ | 28 (0.09)^a^ | 1.472 | 0.230 |
|  |  | (0.162) | (0.123) | (0.102) | Mean=0.134 | |
| **Positive** |  |  |  |  |  |  |
| Bright side | Emphasize the bright side of the case/issue | 184 (0.43)^a^ | 25 (0.14)^b^ | 60 (0.18)^b^ | 42.023 | <0.001 |
| Advantage | General advantage or speciﬁc beneﬁt of the case/issue for any side | 134 (0.31)^a^ | 15 (0.08)^b^ | 34 (0.10)^b^ | 37.375 | <0.001 |
| Future benefit | Promising development or praise the current state of the parent-doctor relationship | 156 (0.36)^a^ | 18 (0.10)^b^ | 25 (0.08)^b^ | 61.322 | <0.001 |
|  |  | (0.367) | (0.107) | (0.255) | Mean=0.231 | |
| **Negative** |  |  |  |  |  |  |
| Dark side | Emphasize the dark side of the case/issue | 75 (0.17)^a^ | 52 (0.29)^b^ | 119 (0.36)^b^ | 17.820 | <0.001 |
| Disadvantage | General disadvantage or speciﬁc cost of the case/issue for any side | 95 (0.22)^ab^ | 30 (0.17)^a^ | 87 (0.26)^b^ | 3.316 | 0.037 |
| Future cost | Problematic future development or criticize the current state of the parent-doctor relationship | 74 (0.17)^a^ | 31 (0.17)^a^ | 87 (0.26)^b^ | 5.690 | 0.003 |
|  |  | (0.189) | (0.207) | (0.297) | Mean=0.230 | |
| **Popular medical science** |  |  |  |  |  |  |
| General knowledge | Scientific knowledge about the disease in the issue | 4 (0.01)^a^ | 5 (0.03)^a^ | 9 (0.03)^a^ | 2.040 | 0.131 |
| Specific knowledge | Issue-related knowledge | 14 (0.03)^a^ | 31 (0.17)^b^ | 33 (0.10)^c^ | 17.552 | <0.001 |
|  |  | (0.021) | (0.099) | (0.064) | Mean=0.051 | |
| *Notes*: a, b, c: different subscripts indicate the existence of a statistically significant difference. | | | | | | |

| Table 1b. Overall landscape of the frames uses by opinion incident types (bootstrapping *n* = 2000) | | | | | | |
| --- | --- | --- | --- | --- | --- | --- |
| Category | Coding description | Violence against physicians | No-death | Patient-death | *F* | *p* |
|  |  | n (mean) | n (mean) | n (mean) | *df*=2 |  |
| **Conflict** |  |  |  |  |  |  |
| Physician-patient conflict | Disagreement between patient/patient's family and doctor | 215 (0.33)^a^ | 0 (0.00)^b^ | 30 (0.14)^c^ | 28.464 | <0.001 |
| Patient-public disagreement | Disagreement between patient and the public opinion | 8 (0.01)^a^ | 0 (0.00)^a^ | 16 (0.08)^b^ | 14.185 | <0.001 |
| Physician-public disagreement | Disagreement between doctor and the public opinion | 23 (0.04)^a^ | 15 (0.22)^b^ | 21 (0.10)^c^ | 22.482 | <0.001 |
| General conflict | Disagreement of two sides or to more than two sides of the problem or issue | 88 (0.13)^a^ | 9 (0.13)^ab^ | 46 (0.22)^b^ | 4.402 | 0.013 |
|  |  | (0.126) | (0.090) | (0.133) | Mean=0.125 | |
| **Cooperation** |  |  |  |  |  |  |
| Cooperation | Cooperation between patient and doctor | 71 (0.11)^a^ | 5 (0.08)^ab^ | 2 (0.01)^b^ | 10.406 | <0.001 |
| Communication | Good communication between patient/ patient's family and doctor | 32 (0.05)^a^ | 2 (0.03)^ab^ | 2 (0.01)^b^ | 3.417 | 0.033 |
| Physician's understanding | Patient’s concerns are well understood by the doctor | 25 (0.04)^a^ | 0 (0.00)^ab^ | 0 (0.00)^b^ | 5.486 | 0.004 |
| Patient's understanding | Doctors’ views are well understood by the patient | 19 (0.03)^a^ | 3 (0.05)^ab^ | 0 (0.00)^b^ | 3.654 | 0.026 |
|  |  | (0.056) | (0.037) | (0.005) | Mean=0.043 | |
| **Attribution** |  |  |  |  |  |  |
| Government | Society/government has the responsibility to solve the problem | 153 (0.23)^a^ | 4 (0.06)^b^ | 52 (0.24)^a^ | 5.630 | 0.004 |
| Physician | Doctors/hospital have the responsibility to solve the problem | 18 (0.03)^a^ | 10 (0.15)^b^ | 51 (0.24)^c^ | 54.724 | <0.001 |
| Patient | Patient or family has the responsibility to solve the problem | 76 (0.12)^a^ | 0 (0.00)^b^ | 13 (0.06)^b^ | 6.575 | 0.001 |
|  |  | (0.125) | (0.070) | (0.182) | Mean=0.134 | |
| **Positive** |  |  |  |  |  |  |
| Bright side | Emphasize the bright side of the case/issue | 226 (0.34)^a^ | 18 (0.27)^a^ | 25 (0.12)^b^ | 20.841 | <0.001 |
| Advantage | General advantage or speciﬁc beneﬁt of the case/issue for any side | 167 (0.25)^a^ | 3 (0.05)^b^ | 13 (0.06)^b^ | 25.323 | <0.001 |
| Future benefit | Promising development or praise the current state of the parent-doctor relationship | 198 (0.30)^a^ | 1 (0.02)^b^ | 0 (0.00)^b^ | 58.019 | <0.001 |
|  |  | (0.299) | (0.110) | (0.060) | Mean=0.231 | |
| **Negative** |  |  |  |  |  |  |
| Dark side | Emphasize the dark side of the case/issue | 188 (0.28)^a^ | 3 (0.05)^b^ | 55 (0.26)^a^ | 9.131 | <0.001 |
| Disadvantage | General disadvantage or speciﬁc cost of the case/issue for any side | 171 (0.26)^a^ | 5 (0.08)^b^ | 36 (0.17)^b^ | 8.570 | <0.001 |
| Future cost | Problematic future development or criticize the current state of the parent-doctor relationship | 159 (0.24)^a^ | 1 (0.02)^b^ | 32 (0.15)^c^ | 12.305 | <0.001 |
|  |  | (0.261) | (0.045) | (0.193) | Mean=0.230 | |
| **Popular medical science** |  |  |  |  |  |  |
| General knowledge | Scientific knowledge about the disease in the issue | 11 (0.02)^a^ | 0 (0.00)^a^ | 7 (0.03)^a^ | 1.834 | 0.160 |
| Specific knowledge | Issue-related knowledge | 37 (0.06)^a^ | 13 (0.19)^b^ | 28 (0.13)^b^ | 12.167 | <0.001 |
|  |  | (0.036) | (0.097) | (0.082) | Mean=0.051 | |
| *Notes*: a, b, c: different subscripts indicate the existence of a statistically significant difference. | | | | | | |

| Table Table 2. Mean and standard deviations of the Weibo frames before/after the pandemic (*n* = 941) | | | | |
| --- | --- | --- | --- | --- |
|  | Pre-pandemic (*n*=742) | Post-pandemic (*n*=199) | Welch’s *t* | *p* |
|  |  |  |  |  |
| Conflict | 0.130 (0.184) | 0.107 (0.147) | 1.872 | 0.062 |
| Cooperation | 0.020 (0.098) | 0.129 (0.205) | −7.324 | <0.001 |
| Attribution | 0.140 (0.213) | 0.109 (0.198) | 1.948 | 0.052 |
| Positive | 0.153 (0.308) | 0.521 (0.444) | −11.009 | <0.001 |
| Negative | 0.206 (0.338) | 0.320 (0.440) | −3.385 | <0.001 |
| Promotion of health knowledge | 0.062 (0.177) | 0.010 (0.086) | 5.820 | <0.001 |
| *Note*: 95% CI refers to 95% confidence interval. | | | | |

| Table 3. Mean and standard deviations of the Weibo frames before/after the epidemic among trilateral opinion leaders | | | | |
| --- | --- | --- | --- | --- |
|  | Pre-epidemic | Post-epidemic | Welch’s *t* | *p* |
| **Media** |  |  |  |  |
| Conflict | 0.175 (0.198) | 0.074 (0.119) | 6.423 | <0.001 |
| Cooperation | 0.032 (0.121) | 0.153 (0.210) | −5.977 | <0.001 |
| Attribution | 0.186 (0.222) | 0.102 (0.187) | 3.953 | <0.001 |
| Positive | 0.251 (0.382) | 0.664 (0.407) | −9.878 | <0.001 |
| Negative | 0.193 (0.335) | 0.179 (0.358) | 0.372 | 0.710 |
| Popular medical science | 0.024 (0.115) | 0.012 (0.101) | 1.051 | 0.294 |
| **General public** |  |  |  |  |
| Conflict | 0.083 (0.156) | 0.080 (0.119) | 0.104 | 0.917 |
| Cooperation | 0.008 (0.072) | 0.080 (0.157) | −2.260 | 0.033 |
| Attribution | 0.132 (0.226) | 0.067 (0.136) | 1.991 | 0.052 |
| Positive | 0.062 (0.185) | 0.387 (0.416) | −3.843 | <0.001 |
| Negative | 0.191 (0.316) | 0.307 (0.396) | −1.391 | 0. 175 |
| Popular medical science | 0.112 (0.224) | 0.020 (0.100) | 3.412 | 0.001 |
| **Physician** |  |  |  |  |
| Conflict | 0.107 (0.172) | 0.193 (0.181) | −3.212 | 0.002 |
| Cooperation | 0.013 (0.079) | 0.099 (0.210) | −2.955 | 0.005 |
| Attribution | 0.094 (0.184) | 0.145 (0.240) | −1.449 | 0.152 |
| Positive | 0.094 (0.229) | 0.258 (0.401) | −2.882 | 0.006 |
| Negative | 0.230 (0.353) | 0.648 (0.464) | −6.224 | <0.001 |
| Popular medical science | 0.076 (0.194) | 0.000 (0.000) | 6.499 | <0.001 |

| Table 4. Mean and standard deviations of the Weibo frames in different types of opinion leaders | | | | | | |
| --- | --- | --- | --- | --- | --- | --- |
| Account type | Conflict | Cooperation | Attribution | Positive | Negative | Popular medical science |
| Media (*n*=430) | 0.146 (0.185)^a^ | 0.066 (0.161)^a^ | 0.162 (0.216)^a^ | 0.367 (0.431)^a^ | 0.189 (0.342)^a^ | 0.021 (0.111)^a^ |
| General public (*n*=182) | 0.082 (0.151)^b^ | 0.018 (0.091)^b^ | 0.123 (0.216)^ab^ | 0.107 (0.255)^b^ | 0.207 (0.329)^a^ | 0.099 (0.213)^b^ |
| Physician (*n*=329) | 0.121 (0.176)^a^ | 0.027 (0.115)^b^ | 0.102 (0.195)^b^ | 0.121 (0.270)^b^ | 0.297 (0.403)^b^ | 0.064 (0.180)^c^ |
| All posts (*n*=941) | 0.125 (0.177) | 0.043 (0.136) | 0.134 (0.210) | 0.231 (0.372) | 0.230 (0.365) | 0.051 (0.163) |
| *F* (2,938) | 8.672 | 11.785 | 7.925 | 60.105 | 8.715 | 16.684 |
| *p* | <0.001 | <0.001 | <0.001 | <0.001 | <0.001 | <0.001 |
| *Notes*: a, b, c: different subscripts indicate the existence of a statistically significant difference. | | | | | | |

| Table 5. Mean and standard deviations of the Weibo frames in different types of physician-patient incident | | | | | | |
| --- | --- | --- | --- | --- | --- | --- |
| Incident type | Conflict | Cooperation | Attribution | Positive | Negative | Popular medical science |
| Incidents of violence against physicians (*n*=661) | 0.126 (0.163)^a^ | 0.056 (0.155)^a^ | 0.125 (0.204)^a^ | 0.299 (0.414)^a^ | 0.261 (0.399)^a^ | 0.036 (0.146)^a^ |
| No-death incident (*n*=67) | 0.090 (0.142)^a^ | 0.037 (0.100)^ab^ | 0.070 (0.148)^a^ | 0.110 (0.187)^b^ | 0.045 (0.141)^b^ | 0.097 (0.199)^b^ |
| Patient-death incident (*n*=213) | 0.133 (0.223)^a^ | 0.005 (0.048)^b^ | 0.182 (0.237)^b^ | 0.060 (0.150)^b^ | 0.193 (0.271)^c^ | 0.082 (0.192)^b^ |
| All posts (*n*=941) | 0.125 (0.177) | 0.043 (0.136) | 0.134 (0.210) | 0.231 (0.372) | 0.230 (0.365) | 0.051 (0.163) |
| *F* (2,938) | 1.561 | 11.628 | 9.412 | 40.076 | 12.467 | 9.383 |
| *p* | 0.210 | <0.001 | <0.001 | <0.001 | <0.001 | <0.001 |
| *Notes*: a, b, c: different subscripts indicate the existence of a statistically significant difference. | | | | | | |
